# Intracoronary artery retrograde thrombolysis combined with percutaneous coronary interventions for ST-segment elevation myocardial infarction complicated with diabetes mellitus: A case report and literature review

**DOI:** 10.3389/fcvm.2022.962127

**Published:** 2022-07-22

**Authors:** Mingzhi Shen, Yichao Liao, Jian Wang, Xinger Zhou, Yuting Guo, Yingqiao Nong, Yi Guo, Haihui Lu, Rongjie Jin, Jihang Wang, Zhenhong Fu, Dongyun Li, Shihao Zhao, Jinwen Tian

**Affiliations:** ^1^Department of Cardiology, Hainan Geriatric Disease Clinical Medical Research Center, Hainan Branch of China Geriatric Disease Clinical Research Center, Hainan Hospital of Chinese PLA General Hospital, Sanya, China; ^2^The Second School of Clinical Medicine, Southern Medical University, Guangzhou, China; ^3^Department of Cardiology, Sixth Medical Center, PLA General Hospital, Beijing, China; ^4^The First Department of Health Care, Second Medical Center of PLA General Hospital, Beijing, China

**Keywords:** diabetes mellitus, myocardial infarction, intracoronary retrograde thrombolysis, thrombosis, reperfusion preconditioning

## Abstract

**Background:**

The management of a large thrombus burden in patients with acute myocardial infarction and diabetes is still a worldwide problem.

**Case presentation:**

A 74-year-old Chinese woman presented with ST-segment elevation myocardial infarction (STEMI) complicated with diabetes mellitus and hypertension. Angiography revealed massive thrombus formation in the mid-segment of the right coronary artery leading to vascular occlusion. The sheared balloon was placed far from the occlusion segment and urokinase (100,000 u) was administered for intracoronary artery retrograde thrombolysis, and thrombolysis in myocardial infarction (TIMI) grade 3 blood flow was restored within 7 min. At last, one stent was accurately implanted into the culprit’s vessel. No-reflow, coronary slow flow, and reperfusion arrhythmia were not observed during this process.

**Conclusion:**

Intracoronary artery retrograde thrombolysis (ICART) can be effectively and safely used in patients with STEMI along with diabetes mellitus and hypertension, even if the myocardial infarction exceeds 12 h (REST or named ICART ClinicalTrials.gov number, ChiCTR1900023849).

## Background

Coronary heart disease and acute myocardial infarction (AMI) are major contributors to global morbidity and mortality. In the meantime, type 2 diabetes (T2DM) is a major risk factor for coronary heart disease and AMI (1–3). Patients with T2DM are at high risk of myocardial infarction and have a poor prognosis after myocardial infarction, especially after STEMI (4). Acute hyperglycemia promotes coagulation and induces platelet aggregation, resulting in increased thrombus burden in STEMI, and a worse prognosis (5). High thrombus burden combined with diabetes remains an important predictor of poor prognosis after STEMI (6).

Primary percutaneous coronary intervention (PPCI) remains the preferred reperfusion strategy for AMI (7). But the management of intracoronary thrombus is still a great challenge in PPCI Thrombus aspiration seems to be a promising strategy in the past years. However, routine thrombus aspiration combined with PPCI has been proved unable to improve clinical outcomes in patients with STEMI. Even in patients with a high thrombus burden, routine thrombus aspiration does not improve outcomes after 1 year and is associated with increased stroke incidence. As such, there is still a long way to go to solve the problem of intracoronary thrombosis, especially in patients with a large thrombus burden.

Here we reported a case of diabetic patients with STEMI. Intracoronary artery retrograde thrombolysis (ICART) combined with PPCI was successfully used to realize micro-perfusion, micro-flow, and micro-opening to produce reperfusion preconditioning, thereby reducing reperfusion injury, no-reflow, and slow blood flow. Slow blood flow has a certain promotion value. ICART has strong application value.

## Case presentation

**FIGURE 1:**
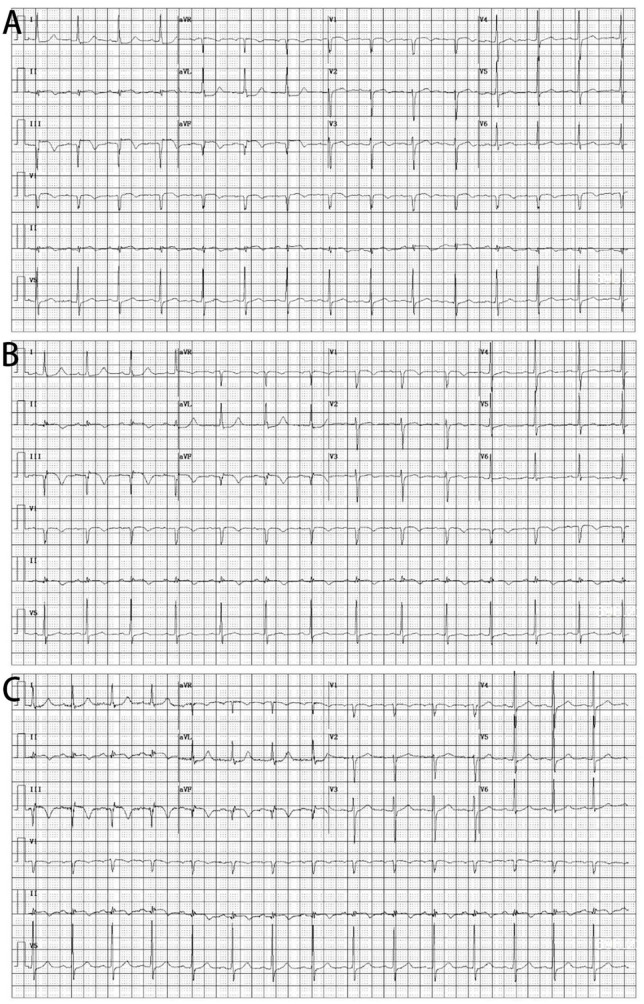
Electrocardiograms. **(A)** In the emergency room. **(B)** After intracoronary artery retrograde thrombolysis (ICART). **(C)** 7 days after ICART.

A 74-year-old woman with sudden chest pain lasting for 17 h was transferred to the emergency department. She had been suffering from diabetes for more than 8 years. Though treated with metformin, the blood glucose was still poorly controlled as evidenced by a fasting blood glucose fluctuating between 8.8 and 10.6 mmol/L, and postprandial blood glucose fluctuating between 9.7 and 13 mmol/L. She also suffered from hypertension for 10 years and took nifedipine controlled-release tablets, and her blood pressure was well controlled. Blood pressure was 145/98 mmHg and pulse rate 103 beats per minute. The electrocardiogram showed ST elevation 0.05–0.2mV in leads II, III, and aVF (Figure 1A). Emergency examination showed that myoglobin reached 359.4 ng/ml, creatine kinase index was 1,094 U/L, creatine kinase isoenzyme index was 167.1 U/L, troponin T was 0.736 ng/ml (the normal reference value: 0–0.1 ng/mL), N-terminal pro-B-type natriuretic peptide (NT-proBNP) was 2235 pg/mL. Cardiac ultrasound showed that the motion of the left ventricular inferior wall was slightly weakened and a small amount of mitral regurgitation. No moist rales were heard on bilateral lung auscultation. The patient was diagnosed with acute inferior ST-segment elevation myocardial infarction, Killip grade 1. The patient had myocardial infarction for 17 h. Although it had been more than 12 h, she still had chest pain, indicating that there was still new myocardial necrosis. Therefore, we chose emergency angiography and PPCI if necessary. Aspirin 300 mg and clopidogrel 600 mg were chewed and swallowed, then the patient bypassed the CCU and went directly to the catheterization laboratory for coronary angiography immediately. The treatment was approved by the Hainan Hospital of PLA General Hospital ethics committee and informed consent was signed.

**FIGURE 2:**
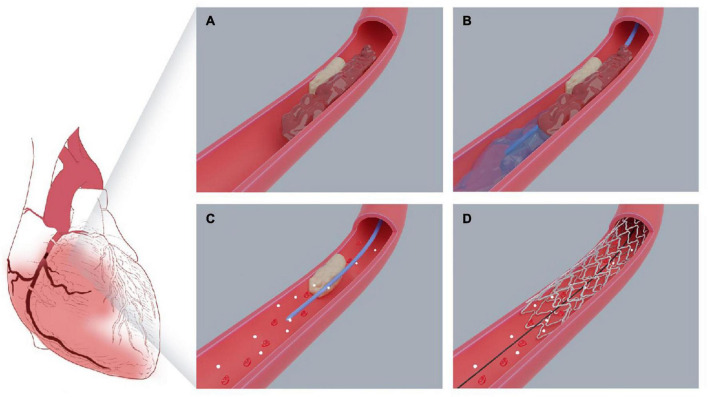
Pattern diagram of intracoronary artery retrograde thrombolysis system. **(A)** The right coronary plaque ruptured, leading to thrombosis. **(B)** The sheared balloon was placed far away from the occluded segment, and the “visual thrombolytic agent” was slowly injected into the occluded site through the sheared balloon. **(C)** The occluded thrombus was completely dissolved and the blood flow recovered. **(D)** The stent was accurately implanted into the lesion.

**FIGURE 3:**
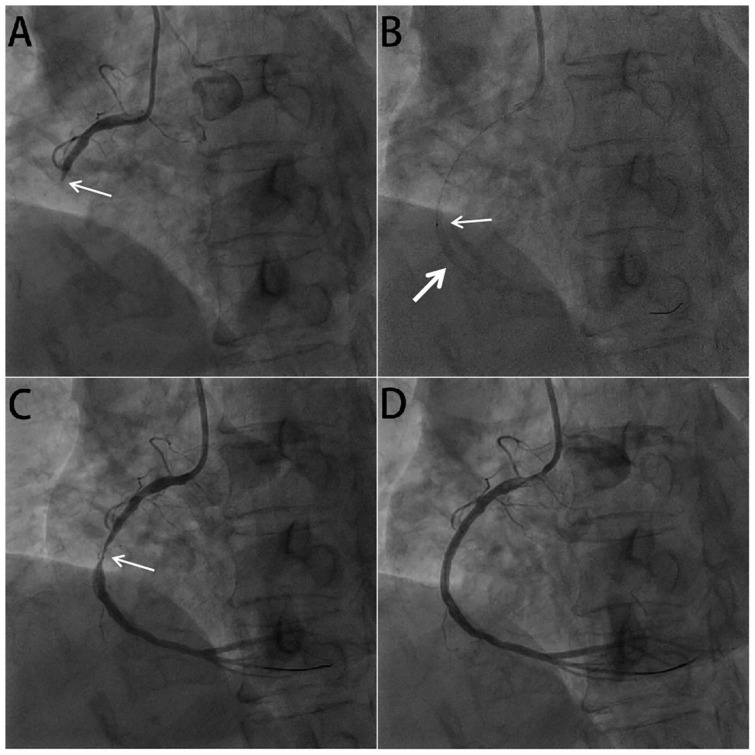
Coronary angiogram in acute right coronary arterial occlusion. **(A)** The basal angiogram showed total occlusion of the right coronary artery distal segment with thrombus image. The arrow showed the occlusion. **(B)** The procedure of intracoronary artery retrograde thrombolysis (ICART) through the sheared balloon. The distal thrombus was gradually dissolved. The fine arrow showed the tip of the sheared balloon, and the coarse arrow indicated the thrombolytic agent with contrast agent to fill the occluded lumen. **(C)** After thrombolysis, the culprit lesion (arrow) in the middle of the right crown was observed. **(D)** A stent was implanted in the RCA to achieve revascularization.

Figure 2 shows the pattern of ICART combined with PPCI in the treatment of this case of acute inferior ST-segment elevation myocardial infarction. Coronary angiography showed that the proximal and middle distal stenosis of the anterior descending artery were 60 and 50%, respectively. Diffuse atherosclerosis from proximal to distal circumflex branch with stenosis. The distal part was the narrowest, and the degree of stenosis was 90%. Mild stenosis was found in the proximal segment of the right coronary artery (RCA), and thrombosis occurred in the middle segment, resulting in vascular occlusion (Figure 3A). Bivalirudin was pumped intravenously for anticoagulation.

Guiding catheter JR4 was connected to the opening of the right coronary artery. A run-through guidewire was used through the culprit artery to the distal end of the occluded RCA. The end of the Sprinter Legend 2 mm × 15 mm balloon was cut off, leaving only the proximal metal ring as a marker. Then the cut balloon was inserted along the run-through guidewire to the distance of the occluded coronary artery (Figure 3B). 100,000 units of urokinase, 15 ml physiological saline, and 5 ml iopromide were mixed to form a 20 ml cocktail, which could be called a visual thrombolytic. Subsequently, 1 mL visualized thrombolytic was bolus-injected through the cut balloon, repeated every 30 s (Figure 3B). During the injection of a visualized thrombolytic agent, the thrombolytic agent mixed with the contrast agent in the occluded lumen could visualize the occluded blood vessel and exert the reverse thrombolytic function through the thrombolytic agent. After thrombolysis with 70,000 u urokinase for 7 min, right coronary angiography showed that the coronary thrombosis disappeared completely and TIMI blood flow recovered to grade 3 (Figure 3C). After accurate positioning, a 2.75 mm × 15 mm stent was directly released by 10 atm pressure. A 3 mm × 12 mm post-dilation balloon was used to dilate at 12 atm for 15 s with less than 5% residual stenosis. Right coronary blood flow returned to TIMI grade 3 (Figure 3D). During the operation, we measured ACT to evaluate the bleeding risk. After 20 min, ACT was 254 s; after 30 min, ACT was 265 s.

The patient’s chest pain symptoms were completely relieved, and the ST segment did not decrease very obviously half an hour after the operation (Figures 1B,C). Bivalirudin was continued for 4 h postoperatively. Tirofiban was applied sequentially for 36 h. The patient regularly took aspirin (100 mg/day), clopidogrel (75 mg/day), rosuvastatin (10 mg/day), bisoprolol (5 mg/day), nicorandil (15 mg/day), and other drugs. For diabetes treatment, the patient received insulin to control blood sugar during the acute phase. On the second postoperative day, the patient started taking metformin hydrochloride (500 mg, tid) regularly to control blood sugar. After treatment, the patient’s fasting blood glucose fluctuated between 6.2 and 7.6 mmol/L and postprandial blood glucose fluctuated between 9.2 and 10.3 mmol/L. The patient had no complications such as hemorrhage and stroke after ICART combined with PCI and was discharged from the hospital 7 days later, and the ST segment did not decrease very obviously.

## Discussion and conclusion

The results of this study demonstrate that ICART combined with PPCI is feasible and can improve myocardial reperfusion in 18-h among patients with STEMI along with diabetes.

Diabetes is seriously affecting public health worldwide, and the number of people with diabetes will continue to increase as obesity and population aging gradually increase (8). The prevalence of adult type 2 diabetes was 8.8% worldwide in 2017, and this proportion is expected to grow to 9.9% by 2045 (9). People with diabetes exhibit a higher risk of cardiovascular complications, while AMI is the primary cause of death in patients with diabetes (10). Diabetic patients with a history of myocardial infarction have a risk of more than 40% of recurrent myocardial infarction (11). Therefore, coronary heart disease (CHD) patients with diabetes need more aggressive treatment to reduce the risk of myocardial infarction compared with CHD in patients without diabetes (12). In addition to being associated with increased cardiovascular risk, T2DM may influence the choice of multiple treatments for CHD, especially myocardial infarction.

Plaque rupture or plaque erosion leads to intracoronary thrombus formation, which in turn causes coronary artery occlusion and ST-segment elevation myocardial infarction (13). PPCI has been shown to have great advantages in establishing effective and early recanalization of infarct-related arteries, reducing major adverse cardiovascular events (MACE), and improving survival (14–16). However, intracoronary thrombus remains the bane of interventional cardiologists (17, 18). Failure to recanalize, suboptimal outcomes, distal embolization, no-reflow, and impaired myocardial perfusion are some of the unresolved difficulties that frequently arise during PCI in patients with a high intracoronary thrombus burden, indicating an unmet need (19, 20). In the high target lesion SYNTAX score lesions receiving balloon predilation, a maximum predilation pressure >10 atm was associated with a higher risk of no-reflow (21).

To reduce thrombus burden in patients with STEMI, a number of methods are used during PPCI, such as distal protection devices (22, 23), thrombus aspiration (6, 24, 25), and glycoprotein IIb/IIIa antagonists (26, 27).

However, several studies, including the AIMI, PROMISE, and EMERALD trials (28), found that distal protection devices were not protective and even detrimental to myocardial perfusion and eventual infarct size.

In patients with a high thrombus burden, routine thrombus aspiration did not improve outcomes at 1 year and was associated with an increased rate of stroke (6, 29). Thrombus aspiration does not appear to be associated with an improvement in clinical outcomes regardless of ischemic time (30). We speculate that this is directly related to reperfusion injury caused by thrombus aspiration, intracoronary artery thrombosis is still a nightmare for interventional cardiologists.

Due to the detrimental effects of acute ischemia/reperfusion injury (I/RI) on the heart, myocardial reperfusion has been referred to as “a double-edged sword” (31). I/RI causes cardiomyocyte death and may in fact cause up to 50% of the final myocardial infarction size (32).

Reperfusion injury is mainly characterized by myocardial stunning, reperfusion-induced arrhythmias, coronary no-reflow phenomenon, and lethal myocardial reperfusion injury (32–34). The above-mentioned problems are the primary problems of opening the occluded blood vessels, that is, how to reduce the thrombus burden and reperfusion injury. Ischemic postconditioning is helpful (35, 36), but due to the heavy thrombus burden, repeated balloon dilation can also lead to thrombus detachment, resulting in slow blood flow or no-reflow. At the same time, during ischemic postconditioning, the culprit’s blood vessels are opened first. In fact, obvious reperfusion has occurred in this process and obvious reperfusion injury occurs. Post-treatment is some remedial measures after reperfusion injury.

In this patient, intracoronary artery retrograde thrombolysis was used. In this process, the thrombolytic agent is administered through the sheared balloon to generate micro-blood flow, micro-opening, and micro-perfusion, which can be called reperfusion preconditioning. This concept has not been proposed yet. Just like a beggar who has been hungry for a long time, if a large amount of meat or broth is suddenly given, it may actually kill the beggar. For patients with STEMI, if the occluded blood vessel is suddenly opened by thrombus aspiration or balloon dilation, it will lead to significant reperfusion injury, malignant arrhythmia, and extensive myocardial necrosis. If the blood flow is fine and opened gradually, the myocardial necrosis will be reduced, and the incidence of malignant arrhythmia will be reduced.

The advantage of intracoronary artery retrograde thrombolysis lies not only in reperfusion preconditioning but also in thrombolysis, showing the distal vascular bed, which is also of great benefit to judging whether the guide wire is in the true cavity, slow blood flow, and no-reflow. At the same time, it can clearly show the lesions, which is very helpful for the accurate selection of stents. On the other hand, the thrombus in the coronary artery is dissolved, which reduces the risk of cerebral infarction caused by thrombus shedding during thrombus aspiration.

This medical record is equivalent to the addition of thrombolytic agents during emergency PCI, but the dosage of urokinase is only 1/15 of the conventional dosage, which is very small. No significant bleeding risk was observed in this patient. Generally speaking, intravenous thrombolysis can be considered for ST-elevation myocardial infarction with an onset of less than 12 h. In this case, 18 h after myocardial infarction, the thrombus can still be dissolved by intracoronary artery retrograde thrombolysis, which reflects that thrombolytic agents have a good effect on the slightly older thrombus as well.

In conclusion, intracoronary reverse thrombolysis is safe and effective for diabetic patients with STEMI with high thrombus load for more than 18 h, and it is an important treatment option. However, based on the application of double suppositories and anticoagulants, the addition of thrombolytics will increase the risk of bleeding relatively. Whether it is suitable for people with high bleeding risk remains to be further observed and more samples will be included. Additionally, the effect on TIMI blood flow needs further observation, whether it can really reduce slow blood flow and no-reflow. In addition, whether it can improve cardiac function and improve ejection fraction remains to be a randomized controlled trial with large sample size.

## Ethics statement

This study has been approved by the Ethics Committee Board of the Chinese PLA General Hospital, Beijing, China. Written informed consent was obtained from the patient for their participation in this case report. Written informed consent was obtained from the individual(s) for the publication of any potentially identifiable images or data included in this article.

## Author contributions

JHW, JW, XZ, YTG, and YN carried out patient management and data collection. MS, DL, SZ, and YG drafted the manuscript and edited the figures. JT, SZ, RJ, HL, and YL performed the angioplasty. JT and ZF critically revised the manuscript for important intellectual content. All authors read and approved the final manuscript.

## Conflict of interest

The authors declare that the research was conducted in the absence of any commercial or financial relationships that could be construed as a potential conflict of interest.

## Publisher’s note

All claims expressed in this article are solely those of the authors and do not necessarily represent those of their affiliated organizations, or those of the publisher, the editors and the reviewers. Any product that may be evaluated in this article, or claim that may be made by its manufacturer, is not guaranteed or endorsed by the publisher.

## Abbreviations

PCI: percutaneous coronary interventions
PPCI: primary percutaneous coronary interventions
STEMI: ST-segment elevation myocardial infarction
TIMI: thrombolysis in myocardial infarction
ICART: intracoronary artery retrograde thrombolysis
AMI: acute myocardial infarction
T2DM: type 2 diabetes
NT-proBNP: N-terminal pro-B-type natriuretic peptide
PLA: People’s Liberation Army
RCA: right coronary artery
CHD: coronary heart disease
MACE: major adverse cardiovascular events
I/RI: ischemia/reperfusion injury.
